# Predicting diagnosis and survival of bone metastasis in breast cancer using machine learning

**DOI:** 10.1038/s41598-023-45438-z

**Published:** 2023-10-25

**Authors:** Xugang Zhong, Yanze Lin, Wei Zhang, Qing Bi

**Affiliations:** 1 Center for Rehabilitation Medicine, Cancer Center, Department of Orthopedics, Zhejiang Provincial People’s Hospital Affiliated to Qingdao University, Qingdao, Shandong People’s Republic of China; 2 Center for Rehabilitation Medicine, Cancer Center, Department of Orthopedics, Zhejiang Provincial People’s Hospital, Affiliated People’s Hospital, Hangzhou Medical College, Hangzhou, 310014 Zhejiang People’s Republic of China; 3 Department of Orthopedics, Taizhou Hospital of Zhejiang Province Affiliated to Wenzhou Medical University, Linhai, Zhejiang 317000 People’s Republic of China

**Keywords:** Oncology, Risk factors

## Abstract

This study aimed at establishing more accurate predictive models based on novel machine learning algorithms, with the overarching goal of providing clinicians with effective decision-making assistance. We retrospectively analyzed the breast cancer patients recorded in the Surveillance, Epidemiology, and End Results (SEER) database from 2010 to 2016. Multivariable logistic regression analyses were used to identify risk factors for bone metastases in breast cancer, whereas Cox proportional hazards regression analyses were used to identify prognostic factors for breast cancer with bone metastasis (BCBM). Based on the identified risk and prognostic factors, we developed diagnostic and prognostic models that incorporate six machine learning classifiers. We then used the area under the receiver operating characteristic (ROC) curve (AUC), learning curve, precision curve, calibration plot, and decision curve analysis to evaluate performance of the machine learning models. Univariable and multivariable logistic regression analyses showed that bone metastases were significantly associated with age, race, sex, grade, T stage, N stage, surgery, radiotherapy, chemotherapy, tumor size, brain metastasis, liver metastasis, lung metastasis, breast subtype, and PR. Univariate and multivariate Cox regression analyses revealed that age, race, marital status, grade, surgery, radiotherapy, chemotherapy, brain metastasis, liver metastasis, lung metastasis, breast subtype, ER, and PR were closely associated with the prognosis of BCBM. Among the six machine learning models, the XGBoost algorithm predicted the most accurate results (Diagnostic model AUC = 0.98; Prognostic model AUC = 0.88). According to the Shapley additive explanations (SHAP), the most critical feature of the diagnostic model was surgery, followed by N stage. Interestingly, surgery was also the most critical feature of prognostic model, followed by liver metastasis. Based on the XGBoost algorithm, we could effectively predict the diagnosis and survival of bone metastasis in breast cancer and provide targeted references for the treatment of BCBM patients.

## Introduction

Currently, breast cancer (BC) is the most common malignant tumor that endangers women’s health. According to 2022 cancer statistics, BC has the highest proportion of malignancies diagnosed in American women, accounting for 31% of new cases, and is also the second leading cause of cancer-related death^1^. With the continuous improvement of BC survival rate, the number of patients with breast cancer metastasis is also increasing^2,3^. Numerous studies have shown that BC exhibits metastatic heterogeneity with distinctive metastatic precedence to diverse organs, thereby resulting in significant differences in prognoses and therapy response of BC patients^4–6^. It is well known that bone is the most common site for distant metastases of BC, with nearly 75% of distant metastasis being bone metastasis (BM)^7^. Given the complexity of metastatic BC therapies, the treatment of BC with bone metastasis (BCBM) is limited to cytotoxic chemotherapies, endocrine therapies, and targeted therapies^8^. Furthermore, although the 5-year overall survival rate of BC patients without metastasis is greater than 80%^9^, distant metastases significantly reduces this rate to only about 25%^10^. Strikingly, the 5-year overall survival rate for BM is even lower, at a measly 22.8%^11^. Studies have also revealed that bone-related events caused by BM, such as bone fracture, hypercalcemia, or spinal cord compression, have a significantly negative impact on the prognosis of BCBM patients^12–14^. Therefore, it is crucial to identify patients who may have bone metastasis and predict their survival rate. This information can guide the subsequent examination, treatment, and management of the patients’ clinical outcomes.

Over the years, tumor-node-metastasis (TNM) staging system, proposed by the American Joint Committee on Cancer (AJCC), and pathological classification, proposed by the World Health Organization (WHO), have been considered as prognostic evaluation systems for BCBM^15,16^. It is worth noting that these systems only incorporate predictors such as tumor infiltrating depth, invasive site, proliferative marker, gene expression assays, and response to neoadjuvant therapy. In recent years, many prediction models for BCBM have been developed, with factors such as age, sex, race, treatment, and grade being the predictors^17,18^. However, these models have specific room for improvement in practicality and accuracy. This study aims at establishing a more accurate clinical model, with as many valid variables as possible.

With regard to model development, although nomogram is currently the most commonly used prediction model, Machine learning is favored by more and more medical workers because of its practicality, innovation and accuracy. This study, a reliable BCBM prediction model is developed through the horizontal comparison of multiple indicators based on demographic characteristics, pathological information, and survival data retrieved from the Surveillance, Epidemiology, and End Results (SEER) database. Furthermore, we developed two web pages as an extension of our research. These web pages enable clinicians to obtain precise and quantitative assessments of the likelihood of bone metastasis and the 5-year survival rate for breast cancer by inputting simple data. Finally, our aim is to stratify the risk of possible bone metastasis and poor prognosis in breast cancer patients. Help clinicians make decisions, reduce the unnecessary medical burden of patients, and greatly improve the quality of patients’ life.

## Materials and methods

### Study population

We collected data from on patients diagnosed with BC between 2010 and 2016 from the SEER database using the SEER*Stat software version 8.3.8.1. Notably, the SEER database, supported by the National Cancer Institute (NCI), covers about 30% of the United States population based on data collected by nearly 18 large cancer registries across the United States^19^. This study did not require approval by the ethics committee, as well as patient consent and agreement because the data was publicly available and there was no specific personal information.

Variables, including age, race, sex, laterality, marital status, grade, AJCC TNM stage, surgery, radiotherapy, chemotherapy, tumor size, bone metastases, liver metastases, lung metastases, brain metastases, breast subtype, ER, PR, and HER2 were extracted from the SEER database. Patients were included according to the following criteria: (1) breast cancer confirmed by biopsy or pathology; (2) age at diagnosis ≥ 20 years; and (3) diagnosed between 2010 and 2016. The following patients were excluded: (1) patients only diagnosed via autopsy or death certificate; (2) breast cancer was not the first primary malignant tumor; and (3) cases with unknown variables. Since our goal is to predict the probability of bone metastasis and the survival outcome of breast cancer patients after metastasis. The focus set by machine learning is bone metastasis and prognosis after bone metastasis. The prognosis was replaced by 5-year survival rate. Considering that some patients were followed for a short period of time and their exact 5-year survival status could not be obtained, we excluded patients who had been followed up for less than 5 years and survived. This was done to maintain rigor of the study and reduce possible selection bias.

### Feature selection and validation strategy

To minimize the negative impact of overfitting, we performed feature selection to remove irrelevant or redundant invalid features. In short, we adopted the analytical thinking commonly used in most articles. Firstly, the univariate analysis was carried out, and the variables with statistically significant differences (P < 0.05) in univariate analysis were incorporated into multivariate analysis, and the variables with statistically significant differences were selected as risk factors. In the diagnostic model, univariable and multivariable logistic regression were performed to screen for risk factors. In the prognostic model, Cox proportional hazards regression were applied to screen for prognostic factors. In addition, to further optimize the model using fivefold cross-validation, we carried out five repeated experiments on the training data and determined the best parameters for each model in the training cohort by grid search method. Finally, the importance of each feature was ranked by Shapley additive explanations (SHAP). In order to prevent the model from being biased and help decision makers understand how to use our model correctly, we need to know the influence of each feature on the final result. To solve this problem, SHAP was developed to analyze the impact of each feature on the predicted results.

The overall dataset collected from the SEER database was randomly divided into two cohorts in a ratio of 7:3, namely training cohort and validation cohort. Metrics such as area under the curve (AUC), accuracy, precision, recall, and F1-score were then used to evaluate the reliability of six machine learning models. Next, calibration curves were constructed and used to compare discrimination between the distinct models. Decision curve analysis (DCA) is a novel algorithm that is commonly used to estimate the net benefit value of a model under different thresholds. Compared to the evaluation indicators mentioned above, DCA could better reflect clinical efficacy of predictive models. After conducting a comprehensive comparison of diverse machine learning models, we chose the model with best prediction ability as the final predictive model. To further confirm the applicability of the selected model, it was evaluated in the validation cohort.

### Machine learning algorithms

Python software was used to build machine learning predictive models. It should be noted that the scikit-learn 0.24.1 package is a very important machine learning library in Python filed, which supports four major machine learning algorithms: classification, regression, reduced dimension and clustering. It also includes three modules: feature extraction, data processing and model evaluation. This retrospective study, mainly included six common machine learning algorithms of this package.

Logistic regression is a generalized linear regression analysis model. Although the dependent variables of logistic regression can be dichotomized or multi-classified, dichotomous ones are more common and easier to explain. Logistic regression is mainly used in epidemiology to explore the risk factors of a disease and predict the probability of occurrence of a disease according to the risk factors.

The decision tree classification algorithm is an instance-based inductive learning method, which can extract a tree-like classification model from the given unordered training samples. The complexity of the predictive classification algorithm is only associated with the number of layers of the decision tree, which is linear, and the data processing efficiency is very high, which is suitable for the occasion of real-time classification. In machine learning, a decision tree is a predictive model which represents a mapping relationship between features and tags. Each node in the tree represents an object, whereas each fork path represents a possible attribute value. Finally, each leaf node corresponds to the value of the object represented by the path from the root node to the leaf node.

Random forest, as the name suggests, establishes a forest in a random way. There are many decision trees in the forest, and there is no correlation between each decision tree in the random forest. It adopts the re-sampling technique of bootstrap to repeatedly and randomly select B samples from the original training sample set with N as the training set, and the other samples as the test set.

Extra tree, an algorithm similar to random forest, uses a series of decision trees to make the final prediction of the class or category to which the data point belongs. However, the difference between extra tree and random forest is that it uses the entire original sample instead of subsampling and replacing the data like a random forest. Another difference is the way nodes are segmented. Although the random forest always chooses the best possible segmentation, the extra tree chooses random segmentation. However, both extra tree and random forest are programmed to optimize the final results.

Bayesian classifier is a general term for a class of classification algorithms, all of which are based on Bayes' theorem. The classification principle of Bayesian classifier is to use a priori probability and Bayesian formula to calculate a posteriori probability, and then select the classification result corresponding to the maximum posterior probability. This study used the Gaussian Naive Bayesian (Gaussian NB) model in the Bayesian classifier.

Extreme gradient boosting (XGBoost) model was developed by the Guestrin group in 2016. Given its fast and accurate properties, the model quickly became famous in machine learning related competitions, and is now widely used in the industrial field. It is an improvement on gradient boosting decision tree (GBDT), which has the remarkable feature of efficiently and flexibly processing missing data and assembling weak predictive models to build accurate predictive models. Notably, XGBoost is more original and better compared with traditional machine learning algorithms.

### Ethical approval and consent to participate

We confirm that all methods were carried out in accordance with relevant guidelines and regulations. The data for this study were obtained from the database. Sample collection, research design was approved by the Ethics Committee of Zhejiang Provincial People's Hospital. We confirming that informed consent was obtained from all subjects and/or their legal guardian(s).

### Statistical analysis

SPSS 25.0 and R 4.0.5 software were used for data description and statistical analysis. Categorical variables are expressed as percentages, whereas continuous variables are expressed as means or medians. Continuous variables conforming to normal distribution were analyzed using Student’s t-test and are presented as mean ± standard deviation. On the other hand, continuous variables not conforming to normal distribution were analyzed using the Mann–Whitney *U* test and are presented as median ± interquartile range. Categorical data were tested using the Chi-square test or Fisher’s exact test. Univariable and multivariable logistic regression analyses were performed to identify risk factors for BM, and variables with *P* < 0.05 in multivariable logistic regression analyses were finally included in the diagnostic model. Similarly, univariate and multivariate Cox proportional hazard regression analysis were performed to identify predictors of prognosis for BM, and variables with *P* < 0.05 in multivariate Cox proportional hazard regression analyses were finally incorporated into the prognostic model. *P* < 0.05 was considered statistically significant.

## Results

### Population features

**Figure 1:**
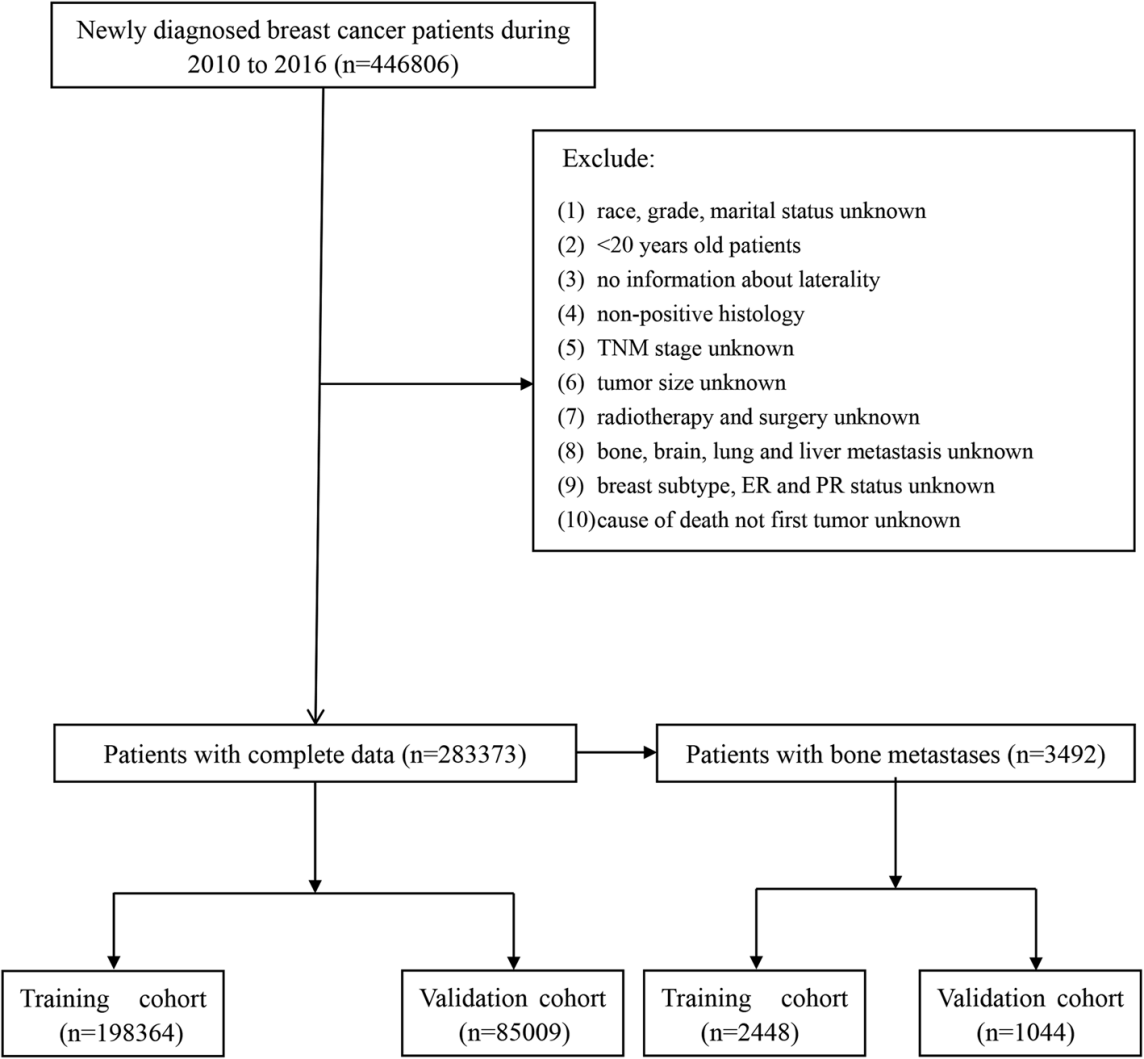
Flow chart of patient screening.

A total of 283,373 BC patients were extracted from the SEER database (198,364 patients in the training cohort and 85,009 patients in the validation cohort) based on the strict inclusion and exclusion criteria, among which 3492 BCBM patients (2448 patients in the training cohort and 1044 patients in the validation cohort) were screened out (Fig. 1). Table 1 shows the demographic and pathological characteristics of BC patients. It was evident that most baseline features were not significantly different between the training and validation cohorts. The results in Table 1 show that the rate of BCBM was about 2.3%, the predominated age was 40–79 years old, and the incidence rate of white people was also much higher than that of other races. Table 2 shows the baseline characteristics of BCBM patients. It was found that the median tumor size of BCBM was obviously larger (44 mm vs 18 mm), the risk of distant metastases was significantly increased, and the proportion of Luminal A BC reached 65.7% (Table 2). All features, both in the diagnostic model and the prognostic model, were analyzed by the Pearson correlation test, and the correlation heat map proved that the variables were independent of each other (Supplementary Fig. 1).

**Table 1:** Demographic and clinicopathological characteristics in breast cancer patients.

Characteristic	Training cohort (n = 198,364)		Validation cohort (n = 85,009)		*P* value
	No. of patients	%	No. of patients	%	
Age					0.485
20–39y	10,782	5.4	4609	5.4	
40–59y	83,651	42.2	35,853	42.2	
60–79y	87,370	44.1	37,592	44.2	
≥ 80y	16,561	8.3	6955	8.2	
Race					0.057
Black	21,976	11.1	9421	11.1	
White	156,913	79.1	67,488	79.4	
Other	19,475	9.8	8100	9.5	
Sex					0.966
Female	196,954	99.3	84,406	99.3	
Male	1410	0.7	603	0.7	
Laterality					0.476
Left	100,382	50.6	43,143	50.8	
Right	97,982	49.4	41,866	49.2	
Marital status					0.589
Married	116,314	58.6	49,811	58.6	
Single	30,877	15.6	13,210	15.5	
Divorced	22,170	11.2	9678	11.4	
Widowed	26,048	13.1	11,030	13	
Separated	2257	1.1	970	1.1	
Unmarried or Domestic Partner	698	0.4	310	0.4	
Grade					0.01
Well differentiated: I	45,730	23.1	19,328	22.7	
Moderately differentiated: II	88,147	44.4	37,509	44.1	
Poorly differentiated: III	63,997	32.3	27,965	32.9	
Undifferentiated; anaplastic: IV	490	0.2	207	0.2	
AJCC T stage					0.641
T1	116,300	58.6	49,704	58.5	
T2	62,675	31.6	27,060	31.8	
T3	12,697	6.4	5391	6.3	
T4	6692	3.4	2854	3.4	
AJCC N stage					0.936
N0	134,065	67.6	57,517	67.7	
N1	47,260	23.8	20,212	23.8	
N2	10,669	5.4	4533	5.3	
N3	6370	3.2	2747	3.2	
Surgery					0.951
No	10,662	5.4	4574	5.4	
Yes	187,702	94.6	80,435	94.6	
Radiotherapy					0.617
No	84,483	42.6	36,119	42.5	
Yes	113,881	57.4	48,890	57.5	
Chemotherapy					0.009
No	114,342	57.6	48,551	57.1	
Yes	84,022	42.4	36,458	42.9	
Tumor size					0.840^#^
Median	18		18		
Range	Nov-28		Nov-28		
Bone metastasis					0.03
No	193,773	97.7	83,154	97.8	
Yes	4591	2.3	1855	2.2	
Brain metastasis					0.76
No	197,953	99.8	84,828	99.8	
Yes	411	0.2	181	0.2	
Lung metastasis					0.078
No	196,215	98.9	84,151	99	
Yes	2149	1.1	858	1	
Liver metastasis					0.358
No	196,593	99.1	84,280	99.1	
Yes	1771	0.9	729	0.9	
Breast subtype					0.073
HR+/HER2− (Luminal A)	146,405	73.8	62,383	73.4	
HR+/HER2+ (Luminal B)	21,392	10.8	9217	10.8	
HR−/HER2+ (HER2 enriched)	8626	4.3	3816	4.5	
HR−/HER2− (Triple Negative)	21,941	11.1	9593	11.3	
ER					0.012
Negative	32,525	16.4	14,264	16.8	
Positive	165,839	83.6	70,745	83.2	
PR					0.014
Negative	52,466	26.4	22,862	26.9	
Positive	145,898	73.6	62,147	73.1	
HER2					0.177
Negative	168,346	84.9	71,976	84.7	
Positive	30,018	15.1	13,033	15.3	

^#^Mann–Whitney U test.

**Table 2:** Demographic and clinicopathological features in breast cancer patients with bone metastasis.

Characteristic	Training cohort (n = 2448)		Validation cohort (n = 1044)		*P* value
	No. of patients	%	No. of patients	%	
Age					0.652
20–39y	174	7.1	76	7.3	
40–59y	985	40.2	402	38.5	
60–79y	1007	41.1	452	43.3	
≥ 80y	282	11.5	114	10.9	
Race					0.986
Black	440	18	186	17.8	
White	1839	75.1	787	75.4	
Other	169	6.9	71	6.8	
Sex					0.248
Female	2416	98.7	1025	98.2	
Male	32	1.3	19	1.8	
Laterality					0.239
Left	1220	49.8	543	52	
Right	1228	50.2	501	48	
Marital status					0.304
Married	1099	44.9	452	43.3	
Single	564	23	259	24.8	
Divorced	343	14	125	12	
Widowed	395	16.1	185	17.7	
Separated	39	1.6	21	2	
Unmarried or Domestic Partner	8	0.3	2	0.2	
Grade					0.597
Well differentiated: I	196	8	78	7.5	
Moderately differentiated: II	1068	43.6	481	46.1	
Poorly differentiated: III	1170	47.8	480	46	
Undifferentiated; anaplastic: IV	14	0.6	5	0.4	
AJCC T stage					0.061
T1	303	12.4	129	12.4	
T2	832	34	402	38.5	
T3	491	20.1	199	19.1	
T4	822	33.6	314	30.1	
AJCC N stage					0.477
N0	561	22.9	256	24.5	
N1	1144	46.7	491	47	
N2	325	13.3	139	13.3	
N3	418	17.1	158	15.1	
Surgery					0.866
No	1606	65.6	688	65.9	
Yes	842	34.4	356	34.1	
Radiotherapy					0.797
No	1386	56.6	596	57.1	
Yes	1062	43.4	448	42.9	
Chemotherapy					0.124
No	1138	46.5	515	49.3	
Yes	1310	53.5	529	50.7	
Tumor size					0.034^#^
Median	44		40		
Range	27–70		27–62		
Brain metastasis					0.137
No	2264	92.5	950	91	
Yes	184	7.5	94	9	
Lung metastasis					0.56
No	1752	71.6	737	70.6	
Yes	696	28.4	307	29.4	
Liver metastasis					0.444
No	1789	73.1	776	74.3	
Yes	659	26.9	268	25.7	
Breast subtype					0.383
HR+/HER2− (Luminal A)	1588	64.9	707	67.7	
HR+/HER2+ (Luminal B)	358	14.6	147	14.1	
HR−/HER2+ (HER2 enriched)	345	14.1	132	12.6	
HR−/HER2− (triple negative)	157	6.4	58	5.6	
ER					0.075
Negative	537	21.9	201	19.3	
Positive	1911	78.1	843	80.7	
PR					0.12
Negative	900	36.8	355	34	
Positive	1548	63.2	689	66	
HER2					0.349
Negative	1933	79	839	80.4	
Positive	515	21	205	19.6	

^#^Mann–Whitney U test.

### Risk and prognostic factors for BCBM

Univariable logistic regression analysis demonstrated that BC patients who underwent surgery had a significantly lower risk of developing BM, suggesting that surgery was the most prominent protective factor (OR = 0.023, 95% CI 0.021–0.024) (Table 3). There were six main risk factors for BM, including grade, T stage, N stage, brain metastasis, lung metastasis, and liver metastasis. The most salient risk factor was brain metastasis (OR = 86.763, 95% CI 70.737–106.884). Furthermore, the multivariable logistic regression analysis revealed that receiving surgery was still a strong protective factor (OR = 0.048, 95% CI 0.044–0.053), and the most statistically significant risk factors were T stage (OR = 6.137, 95% CI 5.294–7.119), N stage (OR = 6.648, 95% CI 5.810–7.603) and liver metastasis (OR = 9.341, 95% CI 8.141–10.723).

**Table 3:** Univariable and multivariable Logistic regression of risk factors of bone metastasis in breast cancer patients.

Characteristics	Univariate analysis		Multivariate analysis	
	OR (95% CI)	*P*	OR (95% CI)	*P*
Age				
20–39y	Reference		Reference	
40–59y	0.661 (0.591–0.741)	< 0.001	0.905 (0.786–1.045)	0.168
60–79y	0.616 (0.551–0.691)	< 0.001	0.953 (0.823–1.106)	0.523
≥ 80y	0.705 (0.611–0.813)	< 0.001	0.527 (0.431–0.645)	< 0.001
Race				
Black	Reference		Reference	
White	0.694 (0.640–0.754)	< 0.001	1.195 (1.071–1.336)	0.002
Other	0.597 (0.525–0.677)	< 0.001	0.822 (0.697–0.967)	0.019
Sex				
Female	Reference		Reference	
Male	2.291 (1.789–2.887)	< 0.001	2.026 (1.493–2.702)	< 0.001
Laterality				
Left	Reference			
Right	0.950 (0.896–1.007)	0.085		
Marital status				
Married	Reference			
Single	1.871 (1.737–2.013)	< 0.001		
Divorced	1.342 (1.221–1.473)	< 0.001		
Widowed	1.302 (1.190–1.422)	< 0.001		
Separated	1.746 (1.368–2.193)	< 0.001		
Unmarried or Domestic Partner	1.131 (0.647–1.820)	0.638		
Grade				
Well differentiated : I	Reference		Reference	
Moderately differentiated : II	2.750 (2.479–3.058)	< 0.001	1.336 (1.182–1.513)	< 0.001
Poorly differentiated : III	3.414 (3.074–3.800)	< 0.001	1.091 (0.954–1.249)	0.205
Undifferentiated; anaplastic :IV	3.877 (2.279–6.150)	< 0.001	0.733 (0.361–1.384)	0.363
AJCC T stage				
T1	Reference		Reference	
T2	5.654 (5.142–6.225)	< 0.001	2.751 (2.461–3.079)	< 0.001
T3	15.907 (14.310–17.698)	< 0.001	3.843 (3.307–4.468)	< 0.001
T4	54.099 (48.922–59.901)	< 0.001	6.137 (5.294–7.119)	< 0.001
AJCC N stage				
N0	Reference		Reference	
N1	6.404 (5.942–6.906)	< 0.001	2.635 (2.399–2.895)	< 0.001
N2	8.215 (7.418–9.089)	< 0.001	4.161 (3.645–4.745)	< 0.001
N3	17.608 (15.960–19.417)	< 0.001	6.648 (5.810–7.603)	< 0.001
Surgery				
No	Reference		Reference	
Yes	0.023 (0.021–0.024)	< 0.001	0.048 (0.044–0.053)	< 0.001
Radiotherapy				
No	Reference		Reference	
Yes	0.534 (0.503–0.567)	< 0.001	1.410 (1.296–1.534)	< 0.001
Chemotherapy				
No	Reference		Reference	
Yes	1.907 (1.797–2.023)	< 0.001	0.762 (0.698–0.836)	< 0.001
Tumor size	1.027 (1.026–1.027)	< 0.001	1.002 (1.000–1.003)	< 0.001
Brain metastasis				
No	Reference		Reference	
Yes	86.763 (70.737–106.884)	< 0.001	4.605 (3.439–6.188)	< 0.001
Lung metastasis				
No	Reference		Reference	
Yes	64.351 (58.746–70.505)	< 0.001	4.718 (4.156–5.357)	< 0.001
Liver metastasis				
No	Reference		Reference	
Yes	80.236 (72.580–88.748)	< 0.001	9.341 (8.141–10.723)	< 0.001
Breast subtype				
HR+/HER2− (Luminal A)	Reference		Reference	
HR+/HER2+ (Luminal B)	1.848 (1.707–1.998)	< 0.001	0.880 (0.789–0.981)	0.022
HR−/HER2+ (HER2 enriched)	1.644 (1.451–1.853)	< 0.001	0.423 (0.358–0.499)	< 0.001
HR−/HER2− (Triple Negative)	0.942 (0.849–1.041)	0.251	0.436 (0.358–0.529)	< 0.001
ER				
Negative	Reference			
Positive	0.977 (0.904–1.057)	0.566		
PR				
Negative	Reference		Reference	
Positive	0.816 (0.765–0.869)	< 0.001	1.121 (1.004–1.253)	0.043
HER2				
Negative	Reference			
Positive	1.802 (1.682–1.930)	< 0.001		

Table 4 shows the statistical results of univariate and multivariate Cox regression analyses. Univariate Cox regression showed that surgery (OR = 0.500, 95% CI 0.457–0.548) was a significant protective factor for the prognosis of BCBM. On the other hand, age, marital status, grade, T stage, brain metastases, lung metastases, liver metastases, and breast subtype were risk factors for the prognosis of BCBM. Moreover, the multivariate Cox regression analysis found that age, race, marital status, grade, surgery, radiotherapy, chemotherapy, brain metastases, lung metastases, liver metastases, breast subtype, ER, and PR were independent predictors of BCBM prognosis. Among them, the most prominent protective factors were surgery (OR = 0.569, 95% CI 0.517–0.628) and ER (OR = 0.482, 95% CI 0.339–0.686). In addition, advanced age, increased tumor grade, and concomitant distant metastases (brain, lung, or liver) worsened the survival outcomes of BCBM patients.

**Table 4:** Univariate and multivariate cox regression of prognostic factors of bone metastasis in breast cancer patients.

Characteristics	Univariate analysis		Multivariate analysis	
	HR (95% CI)	*P*	HR (95% CI)	*P*
Age				
20–39y	Reference		Reference	
40–59y	1.096 (0.922–1.304)	0.3	1.074 (0.899–1.283)	0.428
60–79y	1.409 (1.185–1.674)	< 0.001	1.389 (1.158–1.669)	< 0.001
≥ 80y	1.993 (1.632–2.434)	< 0.001	1.831 (1.459–2.298)	< 0.001
Race				
Black	Reference		Reference	
White	0.731 (0.656–0.814)	< 0.001	0.784 (0.700–0.879)	< 0.001
Other	0.727 (0.604–0.874)	< 0.001	0.849 (0.703–1.028)	0.094
Sex				
Female	Reference			
Male	1.019 (0.715–1.453)	0.915		
Laterality				
Left	Reference			
Right	1.034 (0.951–1.124)	0.432		
Marital status				
Married	Reference		Reference	
Single	1.259 (1.131–1.401)	< 0.001	1.158 (1.035–1.296)	0.011
Divorced	1.289 (1.135–1.467)	< 0.001	1.128 (0.990–1.286)	0.07
Widowed	1.579 (1.401–1.779)	< 0.001	1.185 (1.038–1.354)	0.012
Separated	1.233 (0.884–1.719)	0.218	1.103 (0.787–1.544)	0.569
Unmarried or Domestic Partner	2.109 (1.051–4.232)	0.036	2.075 (1.028–4.189)	0.042
Grade				
Well differentiated : I	Reference		Reference	
Moderately differentiated : II	1.261 (1.067–1.491)	0.007	1.258 (1.062–1.489)	0.008
Poorly differentiated : III	1.572 (1.332–1.856)	< 0.001	1.439 (1.209–1.713)	< 0.001
Undifferentiated; anaplastic :IV	2.131 (1.233–3.683)	0.007	1.437 (0.826–2.500)	0.199
AJCC T stage				
T1	Reference			
T2	1.051 (0.912–1.212)	0.489		
T3	1.187 (1.019–1.384)	0.028		
T4	1.370 (1.189–1.578)	< 0.001		
AJCC N stage				
N0	Reference			
N1	1.015 (0.912–1.130)	0.784		
N2	0.886 (0.766–1.024)	0.099		
N3	0.944 (0.826–1.078)	0.394		
Surgery				
No	Reference		Reference	
Yes	0.500 (0.457–0.548)	< 0.001	0.569 (0.517–0.628)	< 0.001
Radiotherapy				
No	Reference		Reference	
Yes	0.735 (0.675–0.799)	< 0.001	0.809 (0.741–0.884)	< 0.001
Chemotherapy				
No	Reference		Reference	
Yes	0.797 (0.733–0.867)	< 0.001	0.713 (0.645–0.789)	< 0.001
Tumor size	1.003 (1.002–1.004)	< 0.001		
Brain metastasis				
No	Reference		Reference	
Yes	2.124 (1.824–2.474)	< 0.001	1.819 (1.551–2.135)	< 0.001
Lung metastasis				
No	Reference		Reference	
Yes	1.648 (1.504–1.806)	< 0.001	1.286 (1.168–1.415)	< 0.001
Liver metastasis				
No	Reference		Reference	
Yes	1.848 (1.683–2.028)	< 0.001	1.661 (1.499–1.839)	< 0.001
Breast subtype				
HR+/HER2− (Luminal A)	Reference		Reference	
HR+/HER2+ (Luminal B)	1.135 (1.006–1.281)	0.039	0.979 (0.856–1.122)	0.769
HR−/HER2+ (HER2 enriched)	2.141 (1.895–2.418)	< 0.001	0.831 (0.563–1.226)	0.352
HR−/HER2− (Triple Negative)	1.274 (1.072–1.514)	0.006	0.429 (0.285–0.646)	< 0.001
ER				
Negative	Reference		Reference	
Positive	0.565 (0.511–0.624)	< 0.001	0.482 (0.339–0.686)	< 0.001
PR				
Negative	Reference		Reference	
Positive	0.619 (0.568–0.676)	< 0.001	0.702 (0.621–0.793)	< 0.001
HER2				
Negative	Reference			
Positive	1.055 (0.953–1.169)	0.304		

### Predictive performance of the machine learning models for diagnosis and prognosis

#### Diagnostic model

**Figure 2:**
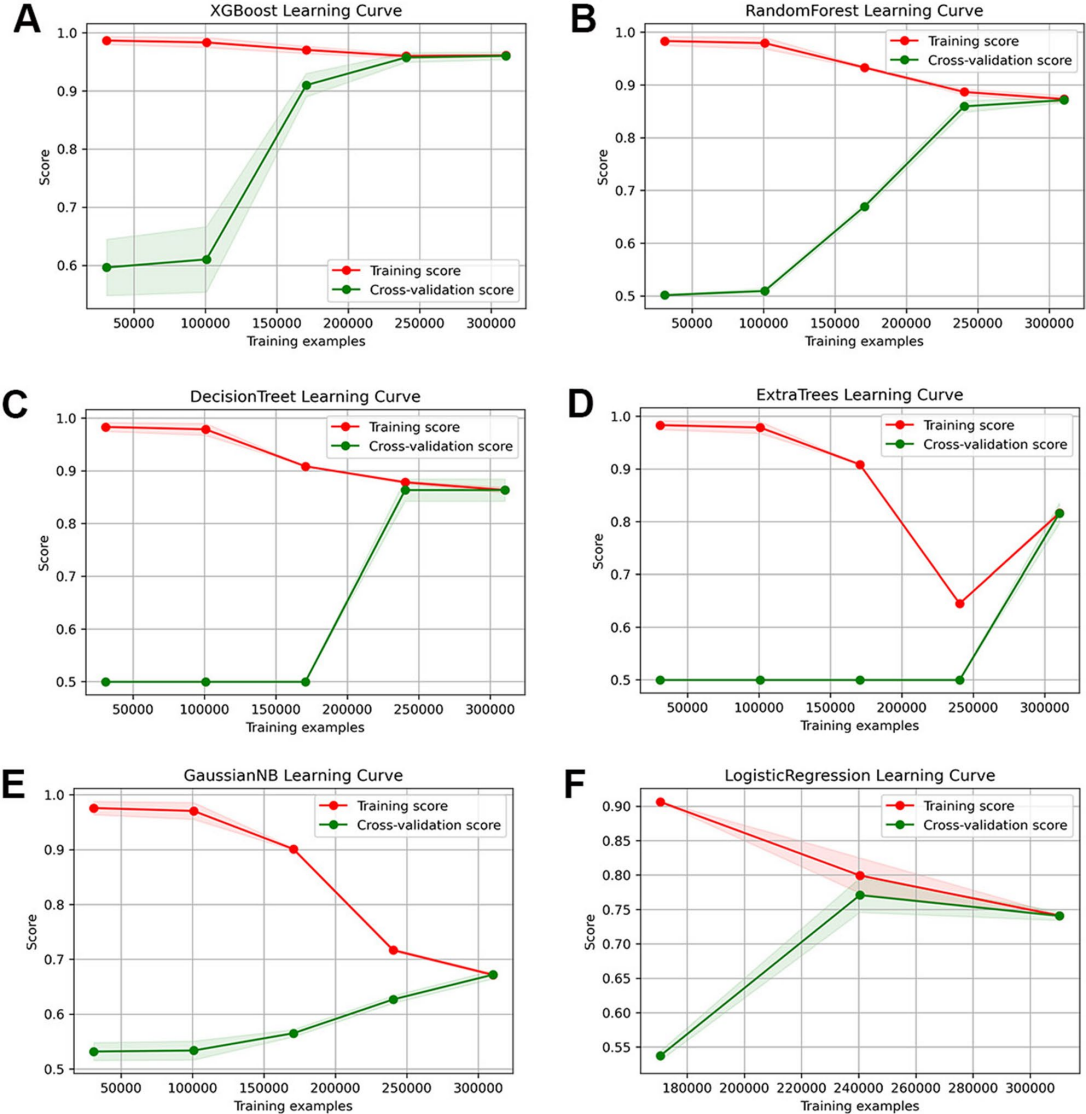
Learning curves of models with training data. (**A**) XGBoost; (**B**) Random Forest; (**C**) Decision Trees; (**D**) Extra Trees; (**E**) Gaussian NB; (**F**) Logistic regression.

**Figure 3:**
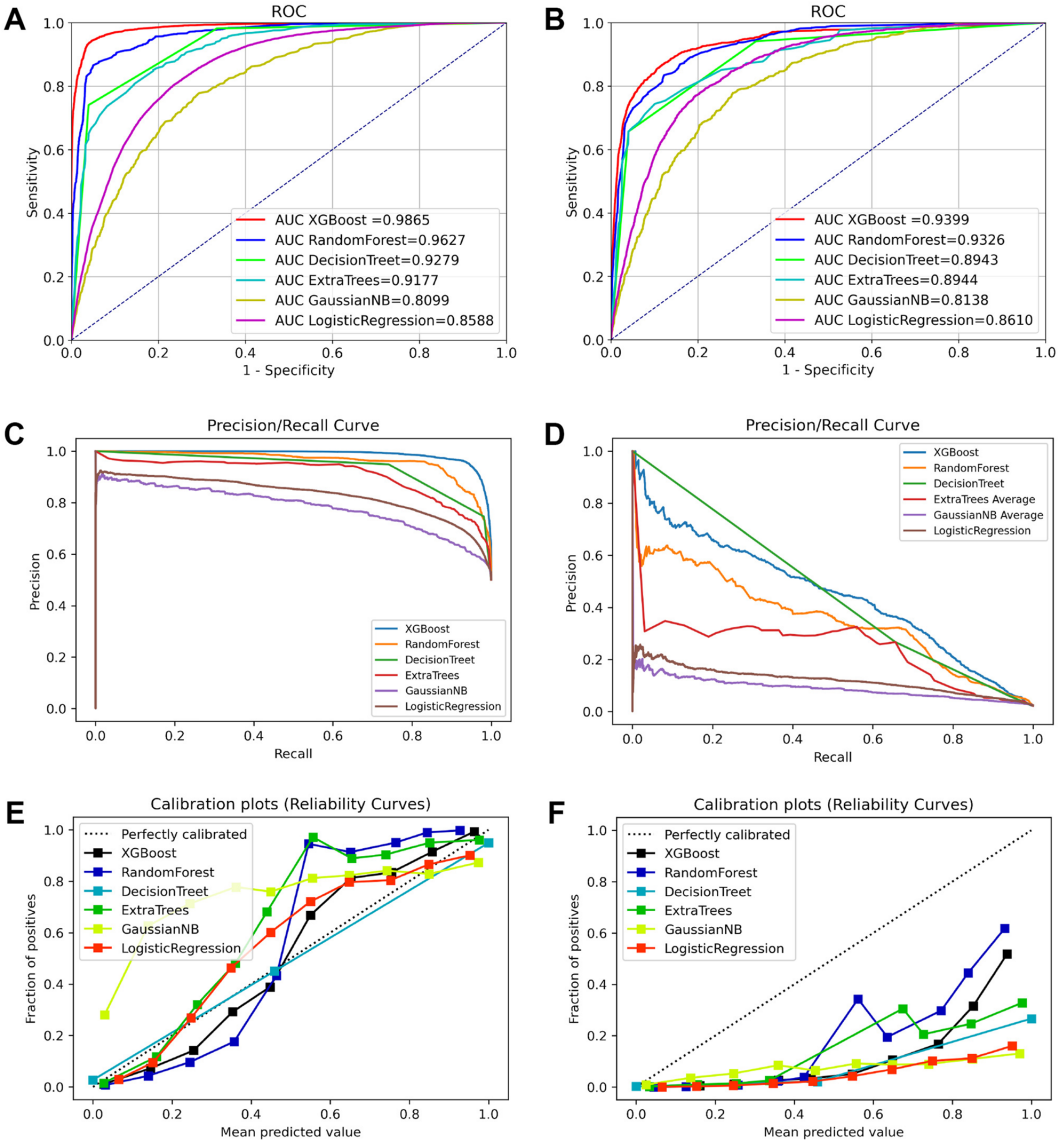
ROC curves of diagnostic models developed by training cohort (**A**) and validation cohort (**B**); PR curves of models developed by training cohort (**C**) and validation cohort (**D**); calibration curves of models developed by training cohort (**E**) and validation cohort (**F**).

Herein, six machine learning models were developed and evaluated through learning curves, AUC, PR curves, and calibration curves. With the continuous increase of learning samples, the learning ability of the model tended to be stable, and finally XGBoost stood out from all models (Fig. 2). Figure 3 shows the evaluation curves of the six models. Considering the superiority of machine learning, the AUC values of all models exceeded 0.80, and the AUC values of XGBoost reached an astonishing 0.987 and 0.940 in the training cohort and the validation cohort, respectively. However, given that the distribution of positive and negative events in the dataset was uneven, AUC alone was not sufficient to explain the performance of the model. Therefore, the PR curve was generated to make up for the inadequacy of the receiver operating characteristic (ROC) curve, thereby further evaluating the strengths and weaknesses of the model. From Fig. 3C,D, it is evident seen that the average precision of the accuracy of the XGBoost model was higher than that of other models. Finally, the calibration curve was drawn to compare the discrimination of each model, with results showing that the XGBoost model still maintained the best state. Table 5 summarizes the evaluation index values ​​of all models. Collectively, these results showed that XGBoost had the most outstanding comprehensive performance, with the highest AUC (0.987), accuracy (0.947), precision (0.948), recall (0.947), and F1-score (0.947).

**Table 5:** The performance of diagnostic models on six ML algorithms.

Models	Training cohort					Validation cohort				
	AUC	Accuracy	Precision	Recall	F1-score	AUC	Accuracy	Precision	Recall	F1-score
XGBoost	0.987	0.947	0.948	0.947	0.947	0.939	0.938	0.978	0.938	0.954
RF	0.963	0.888	0.889	0.888	0.888	0.932	0.846	0.976	0.846	0.899
DT	0.928	0.85	0.868	0.85	0.849	0.894	0.953	0.976	0.953	0.962
ET	0.918	0.821	0.839	0.821	0.819	0.894	0.917	0.975	0.917	0.941
GaussianNB	0.81	0.669	0.718	0.669	0.649	0.813	0.894	0.967	0.894	0.926
LR	0.859	0.769	0.775	0.769	0.768	0.861	0.797	0.973	0.797	0.868

*XGBoost* Extreme gradient boosting, *RF* Random Forest, *DT* Decision Tree, *ET* Extra Tree, *GaussianNB* Gaussian Naive Bayesian, *LR* Logistic Regression.

#### Prognostic model

**Figure 4:**
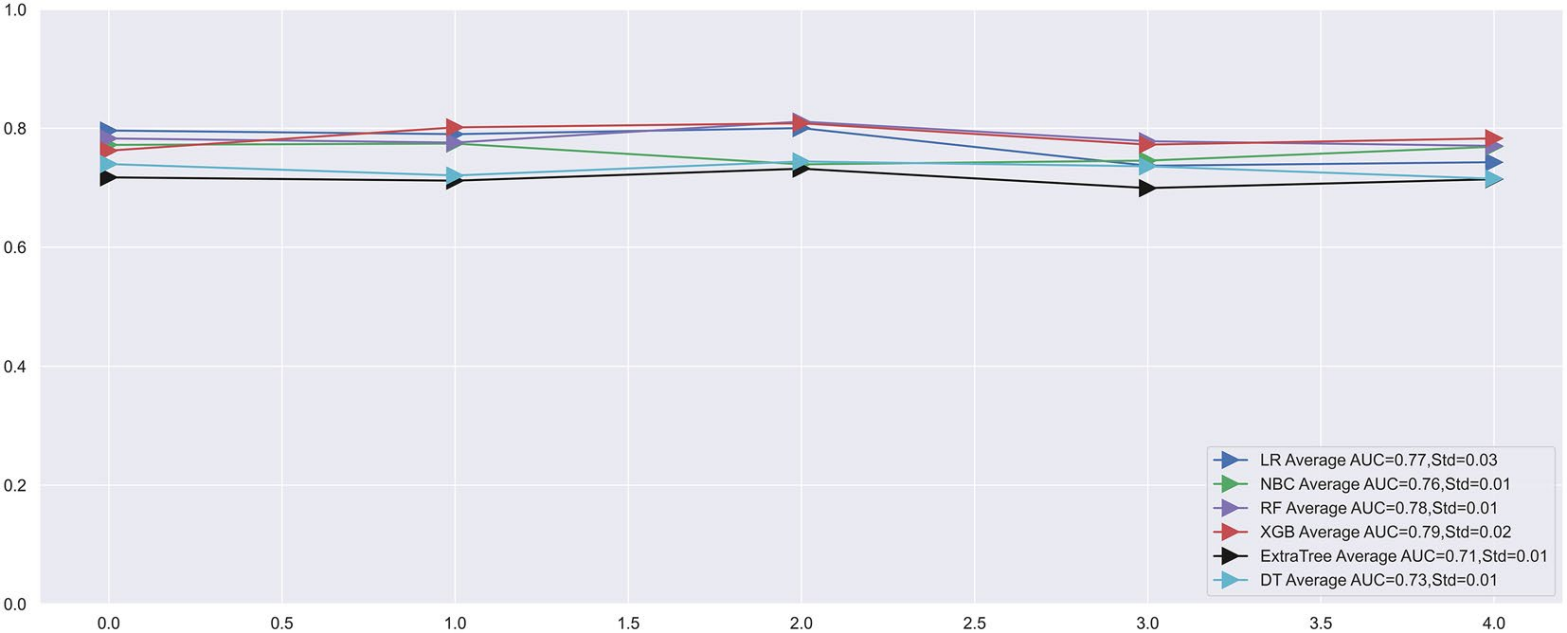
Ten-fold cross-validation results of the six machine learning models in the training group.

**Figure 5:**
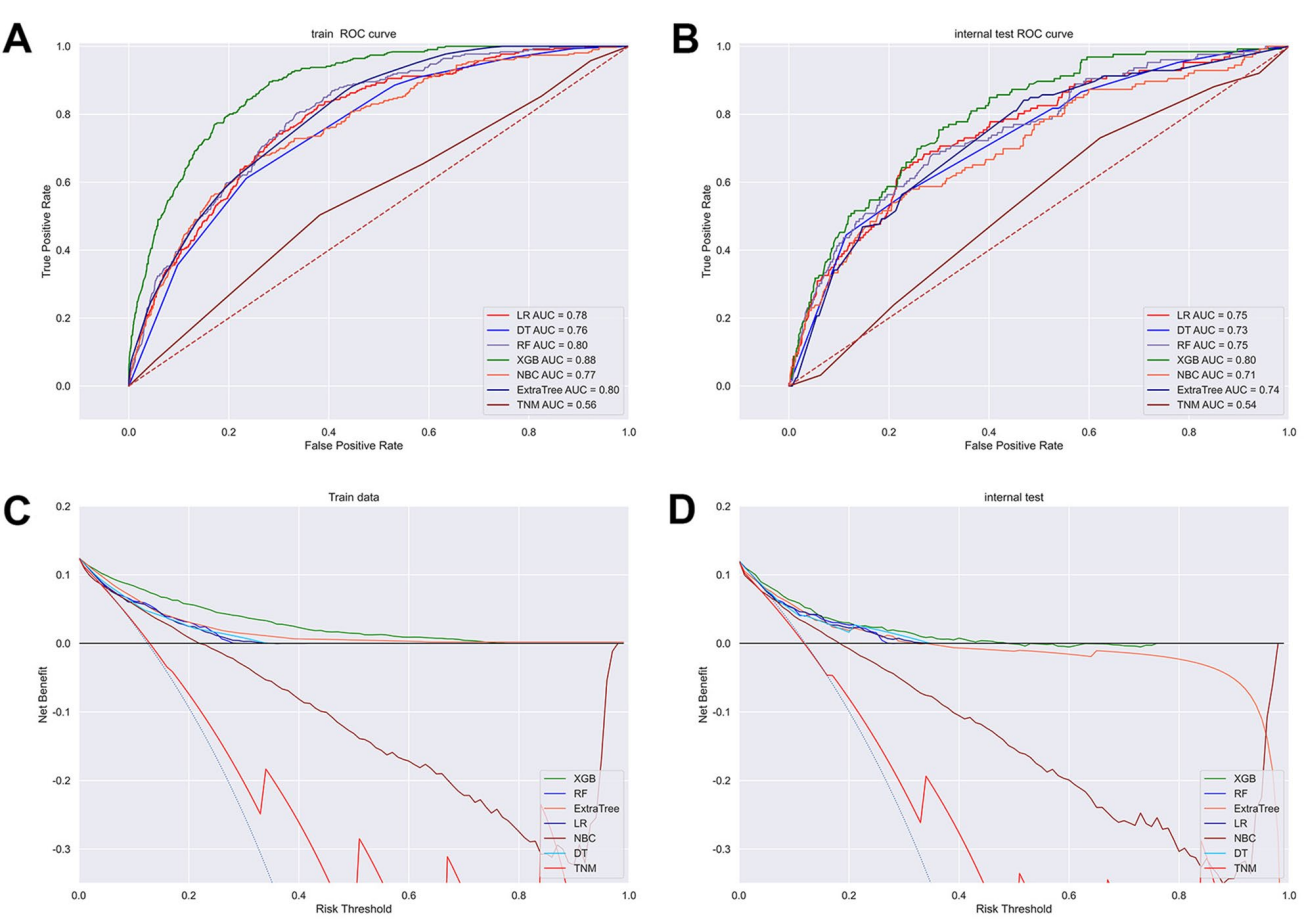
The ROC curves of prognostic models based on machine learning in training set (**A**) and validation set (**B**). The decision curves of prognostic models based on machine learning in training cohort (**C**) and validation cohort (**D**).

**Figure 6:**
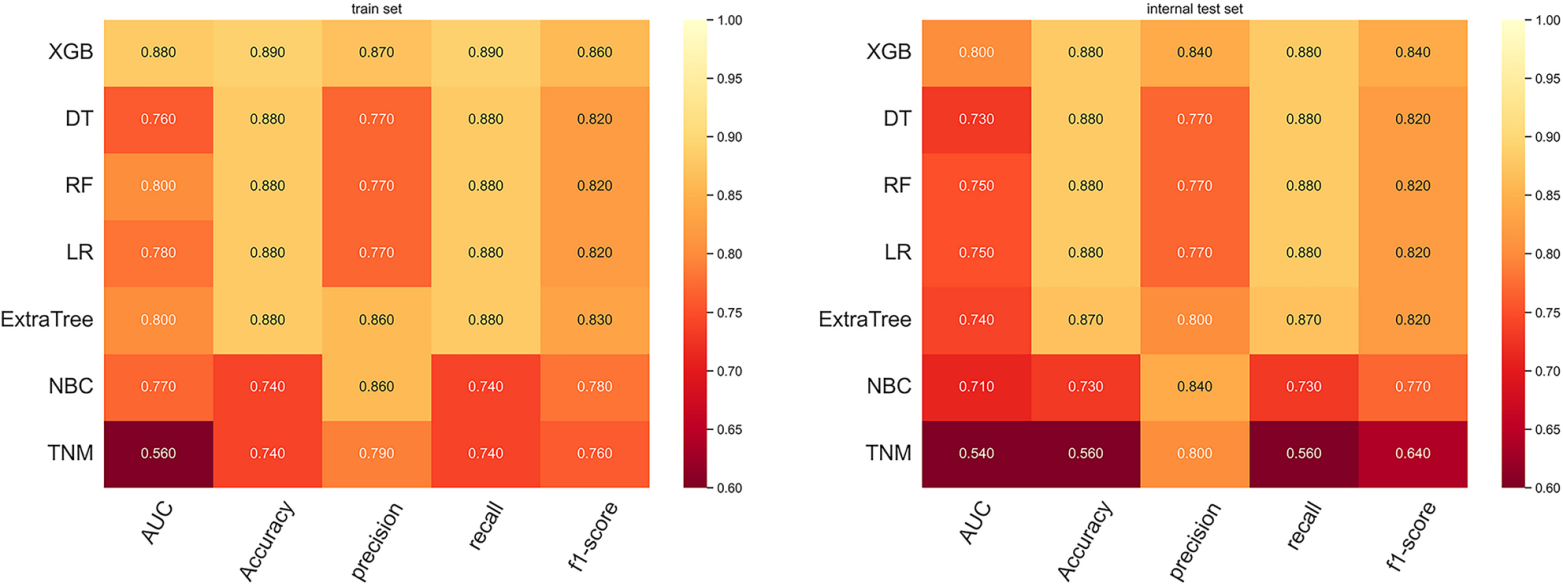
Prediction performance of seven models.

Five-fold cross-validation was applied to evaluate the performance of the machine learning prediction model, and the results obtained after 5 repetitions and the average ROC curve of the different generated ROC curves were used as the evaluation metric. Based on the model we built, the XGBoost model performed the best in five-fold cross-validation with an average AUC of 0.79 (Fig. 4). It was evident that the XGBoost model displayed excellent accuracy both in the training set and validation set. Furthermore, DCA suggested that the XGBoost model exhibited a better clinical application value in both training set and validation set, and it performed significantly better than the traditional AJCC staging system (Fig. 5). In the training cohort, the XGBoost model scored the highest with an AUC of 0.880, an accuracy of 0.890, a precision of 0.870, a recall of 0.890 and a F1-score of 0.860. The XGBoost model also scored the highest in the validation cohort, with an AUC of 0.800, an accuracy of 0.880, a precision of 0.840, a recall of 0.880, and a F1-score of 0.840 (Fig. 6). Finally, a heatmap was generated to indicate the prediction effect of the distinct models (Supplementary Fig. 2).

### Characteristic importance in the machine learning models

**Figure 7:**
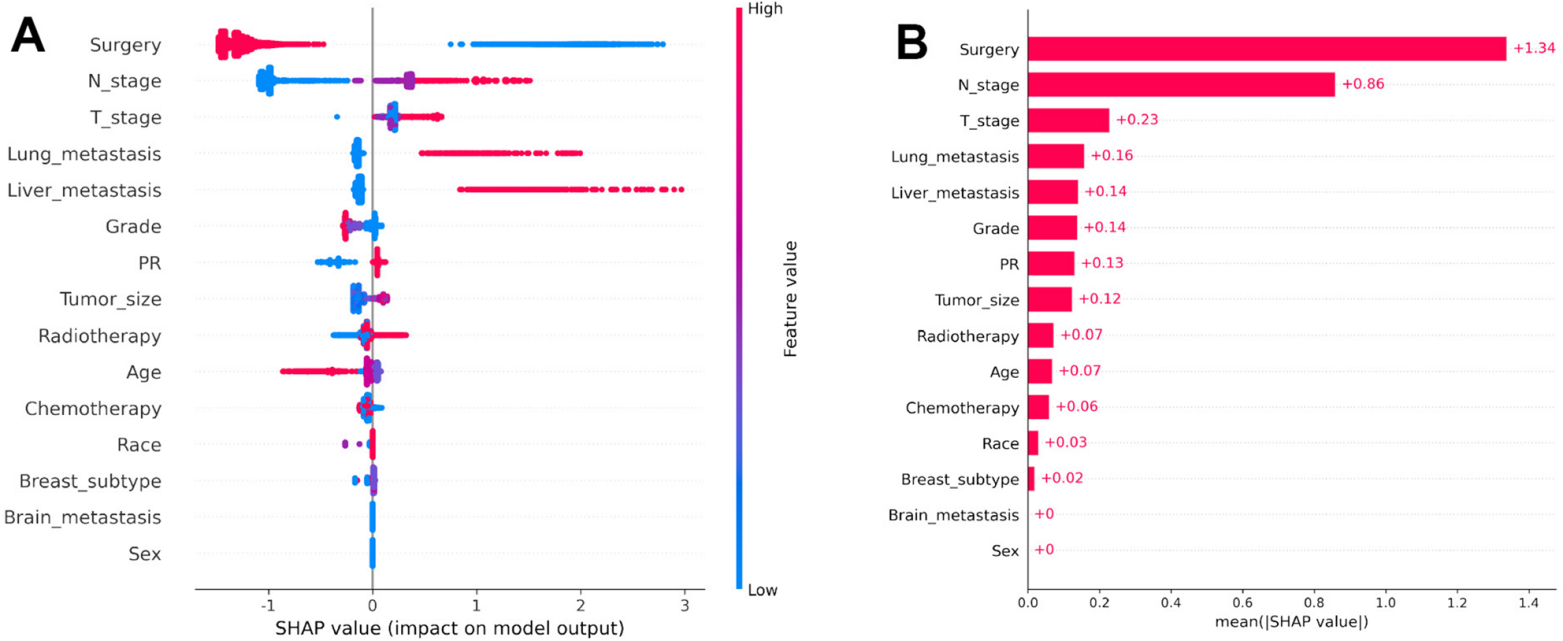
Feature importance ranking by SHAP values in diagnostic model based on the XGBoost algorithm. (**A**) The features are sorted according to the sum of the SHAP values of all patients, and SHAP values are used to represent the distribution of the influence of each feature on the output of the XGBoost model. Red indicates that the value of the feature is higher, whereas blue indicates that the value of the feature is lower. The X-axis represents the effect of SHAP values on the output of the model. The higher the value of X-axis, the greater the likelihood of delayed mitigation. (**B**) The standard bar chart is drawn and sorted using the average absolute value of each feature shape value in the XGBoost model.

**Figure 8:**
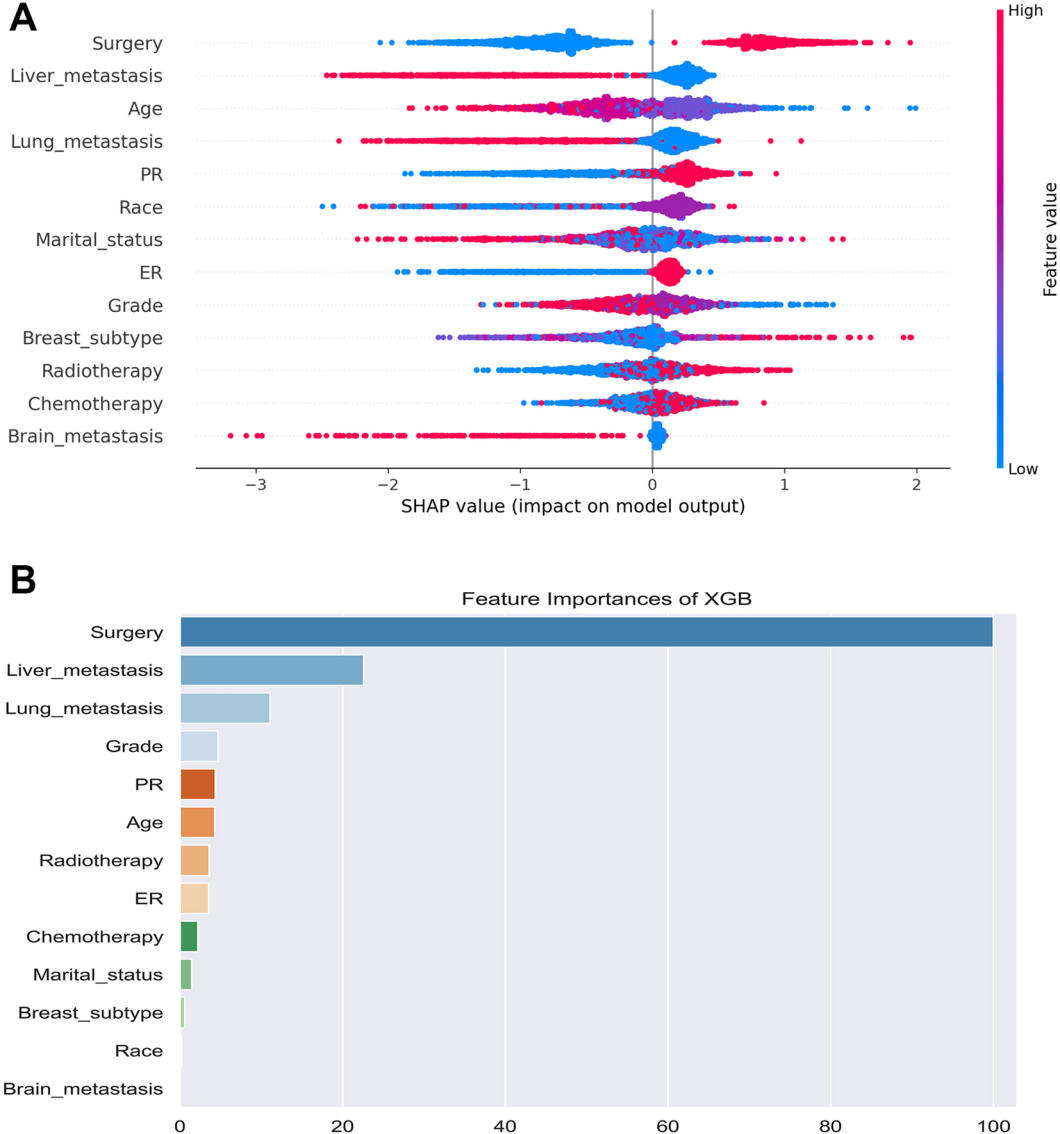
Feature importance ranking by SHAP values in prognostic model based on XGBoost algorithm. (**A**) The features are sorted according to the sum of the SHAP values of all patients, and SHAP values are used to represent the distribution of the influence of each feature on the output of the XGBoost model. Red indicates that the value of the feature is higher, whereas blue indicates that the value of the feature is lower. The X-axis represents the effect of SHAP values on the output of the model. The higher the value of X-axis, the greater the likelihood of delayed mitigation. (**B**) The standard bar chart is drawn and sorted using the average absolute value of each feature shape value in the XGBoost model.

The SHAP diagram was utilized to more intuitively express the importance of each feature of the model. According to the multivariable logistic regression and multivariate Cox proportional hazards regression results, we included 15 and 13 features in the diagnostic and prognostic models, respectively. The SHAP plot was then used to rank these important features, indicating the degree of influence of different features on diagnosis and prognosis. From Fig. 7, it is not difficult to find that the higher the SHAP value of a feature, the greater the probability of BM in BC patients. Blue indicated that the eigenvalues were small, purple indicated that the eigenvalues were close to mean value, and red indicated that the eigenvalues were large. Taking the most striking feature in the figure as an example, we found that the incidence of BM was significantly reduced in patients who underwent surgery. Figure 8 shows that surgery still remained the most important feature, with results indicating that the 5-year survival rate of patients who underwent surgery was extremely increased.

## Discussion

According to the latest cancer statistics^1^, BC has replaced lung cancer as the most common cancer in the America. Consequently, BC treatment and handling the corresponding complications have brought a heavy medical burden to the society, which is a major problem associated with human cancer. In particular, the quality of life and survival rate of BC patients are significantly reduced after they develop BC. This is mainly attributed to the occurrence of skeleton-related events (SREs) after BM. Studies have revealed that the severe vertebral invasion, pathological fractures, bone pain, and other SREs pose a serious threat to the prognosis of patients with BM^20,21^. A previous survey found that the cumulative incidence of SREs in patients with BM is about 45.1%^22^. In the present study, the incidence of BM in BC patients was about 2.3%, which reflects the difficulty of diagnosing patients with BM and the harmfulness of BM. Therefore, it is necessary to effectively screen patients who are prone to develop BM and have poor prognosis after BM. Bone scan combined with CT is the gold standard for detecting BM, and is also the preferred method recommended in current guidelines^23–25^. A recent European prospective study showed that [^18^F] FDG PET/MRI and MRI were significantly better than CT or bone scintigraphy for the detection of BM in newly diagnosed BC patients^26^. However, these tests have some drawbacks, such as radiation damage and high cost, and not all patients are willing to undergo BM testing. Thus, to more effectively address these issues, this study aimed at developing two facile clinical models for early detection of high-risk BCBM patients and prediction of BCBM prognosis.

With the rapid development of artificial intelligence technology, machine learning is increasingly being applied in the field of biomedicine, and it also has great potential in future clinical practice^27,28^. In 2022, an article published in the journal Nature by Stephen-John Sammut et al. presented a study encompassing clinical information, pathology, genomics, and transcriptomics of 168 patients with breast cancer undergoing chemotherapy. They successfully predicted the complete response of chemotherapy patients using a multi-group machine learning approach (AUC = 0.87)^29^. This groundbreaking study demonstrates the significant medical value that mature machine learning models can offer in clinical practice, enabling the provision of more accurate assistance to doctors and patients through alternative methods.

Nevertheless, despite significant advancements in building and utilizing various models, there is still considerable scope for improvement. Li et al. developed a deep learning algorithm that predicts bone metastasis in breast cancer by incorporating MRI radiological features from 96 cases of metastatic breast tumors and 192 cases of non-metastatic breast tumors. The predictive performance of the model is evaluated using statistical morphology and grayscale characteristics, employing metrics such as AUC, sensitivity, and specificity^30^. Nonetheless, due to the high demand for front-end MRI images and data, this model cannot be widely adopted. Thio et al. integrated survival data from thousands of cancer patients with extensive bone metastasis to develop a survival prediction model^31^. However, the majority of the data utilized for model development and verification originates from laboratory sources, including biochemical data, blood routine data, protease data, and more. While accurately predicting the survival rate, it also imposes more stringent demands on the types of data used. Our machine learning model is specifically designed to predict the occurrence of bone metastasis in breast cancer patients and prognosticate patients with bone metastasis. All the parameters required for the model are derived from routine clinical practice, making them more accessible than specific images or laboratory data. This model can also be utilized by hospitals in remote areas or by junior clinicians to guide the comprehensive treatment planning of breast cancer patients, enabling early intervention to prevent and address potential clinical adverse events. Secondly, it employs multiple strategies, such as preventing overfitting and utilizing shrinkage and column subsampling techniques, to enhance algorithmic generalization and learning speed. The XGBoost algorithm, which has demonstrated high accuracy and ease of use in numerous studies^32–34^, is referenced in this model.

This study used univariable and multivariable logistic regression analyses to screen fifteen independent risk factors, including age, race, sex, grade, T stage, N stage, surgery, radiotherapy, chemotherapy, tumor size, brain metastasis, lung metastasis, liver metastasis, breast subtype, and PR. According to the order of importance of the SHAP diagram, the features that contributed prominently were surgery, N stage, and T stage. Next, univariate and multivariate Cox proportional hazards regression analyses were applied to screen thirteen independent prognostic factors, including age, race, marital status, grade, breast subtype, surgery, radiotherapy, chemotherapy, brain metastases, liver metastases, lung metastases, ER, and PR. All features were also ranked by importance, with results showing that surgery, liver metastases, and lung metastases were the three factors strongly associated with prognosis. However, some features that were considered meaningful in the multivariable logistic regression analysis and multivariate Cox proportional hazards regression analysis had a SHAP value of zero in importance ranking. This may further reflect the superiority of machine learning. Specifically, it can better eliminate unnecessary features unlike traditional linear regression analysis, which has the problem of overfitting. Machine learning enables us to obtain more accurate predictive models by continuously improving operational efficiency and self-improvement.

This study found that BC patients who did not undergo surgery were at high risk of developing BM. Yao et al.^17^ also suggested that surgery was an independent risk factor for BCBM. Despite the hazard of radiation damage, we still recommend bone scans to examine BM in unoperated BC patients. We also found that T stage and N stage were strong predictors of BM. Studies have demonstrated that the increase of T and N stages of malignant tumors indicates the increase of tumor volume, and the expansion of the degree and extent of involvement of adjacent tissues and lymph nodes, which are the manifestations of further development of malignant tumors^35,36^. It is well known that the TNM staging system proposed by the AJCC is a widely used prognostic system^37^. However, previous studies have shown that the accuracy of using the TNM staging system alone to predict metastases is not high, and thus researchers often obtain better prediction results through comprehensive analysis of multiple factors^38,39^. Interestingly, surgery was also the most prominent feature with regard to prediction of BCBM prognosis. Although metastatic BC remains an incurable disease, surgery to remove the primary tumor is associated with improved survival in patients with distant metastatic BC at diagnosis. One study reported that patients who underwent primary surgery had significantly longer median survival than those who did not, and primary tumor resection for primary BCBM reduced the risk of death by approximately 40%^40^. A randomized controlled trial conducted in Turkey found that the 3-year OS was similar in patients with and without primary BC surgery. However, at a median follow-up of 5 years, patients who underwent surgery had a prolonged median OS by approximately 9 months^41^. In addition, a trial conducted in India, revealed that the OS of patients with de novo metastatic BC was not improved after surgery for their primary BC^42^. Scholars in Europe concluded that surgical treatment of the primary tumor in patients with de novo metastatic BC could not benefit majority of them^43^. A retrospective study by Gong et al.^44^ identified surgery as an independent prognostic factor for BCBM, which is consistent with our findings. Therefore, whether the primary tumor of BCBM should be operated is still controversial, which calls for further multicenter prospective studies for verification. Liver and lung metastases play an important role in predicting the prognosis of BCBM. This study found that BCBM patients with liver metastasis or lung metastasis had a poor prognosis, and their 5-year survival rate was lower than that of other types of BCBM patients. We comprehensively considered all meaningful features to predict the prognosis of BCBM and achieved good predictive performance.

The ultimate purpose of building models is to be more convenient for clinical application and help clinicians make decisions. Consequently, based on the XGBoost algorithm, we built two accessible online websites (https://share.streamlit.io/lry4000/bone_metastasis/main) and (https://share.streamlit.io/lry4000/sc5_new/main ). Specifically, a streamlined web page structure enables users to input data more efficiently. The clinical parameters mentioned in the article are displayed on the right side of the webpage, allowing users to input corresponding clinical data based on the actual condition of the patients. The system will instantly generate the predicted probability of bone metastasis for the patient. The results can be presented in various formats and shared with a broader range of clinical participants. The second web page, which predicts the survival rate, follows a similar usage process.

There are some limitations in our study. First, this is a multicenter retrospective study involving only patients from the United States, and thus it inevitably suffers from selection bias. Therefore, there is a need for external data from other countries to validate the reproducibility of our results. Second, although our model achieved good clinical performance on the basis of SEER database, it is essential to further confirm the reliability of the model through prospective studies. Third, the SEER database does not include blood routine, biochemical indicators, and Charlson Comorbidity Index (CCI), which may lead to the model missing some important features.

## Conclusion

This study introduced the XGBoost-based machine learning model, for the first time, to construct the diagnosis system and survival prediction system for BCBM patients. We sorted the importance of different features using the demographic characteristics and pathological indicators screened from the SEER database. Furthermore, ROC curves, learning curves, precision curves, calibration plots, and decision curves were used to evaluate performance of the model, and an external verification cohort was established to further verify the model. Finally, we have developed two sample and convenient network applications for helping clinicians better achieve clinical decision-making.

### Supplementary Information

Supplementary Figure S1.

Supplementary Figure S2.

Supplementary Legends.

## Data Availability

The datasets generated during and/or analyzed during the current study are available from the corresponding author on reasonable request.
