# Supplementary figures and images for: Predicting diagnosis and survival of bone metastasis in breast cancer using machine learning
Source: Sci Rep. 2023 Oct 25;13:18301. doi: 10.1038/s41598-023-45438-z (PMC10600146; doi:10.1038/s41598-023-45438-z)

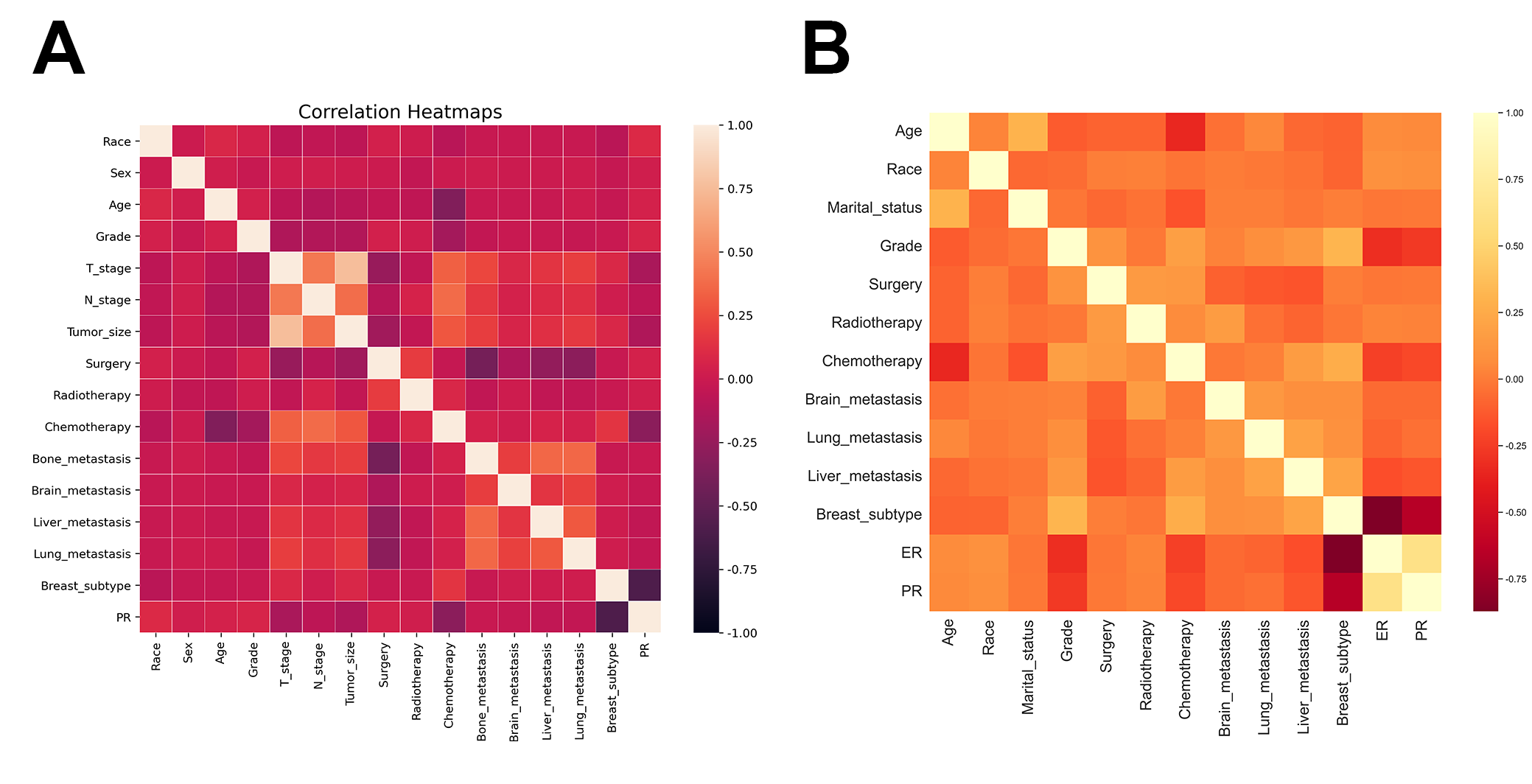

Supplement: Supplementary file 1 — Supplementary Figure S1. [file 41598_2023_45438_MOESM1_ESM.tif]

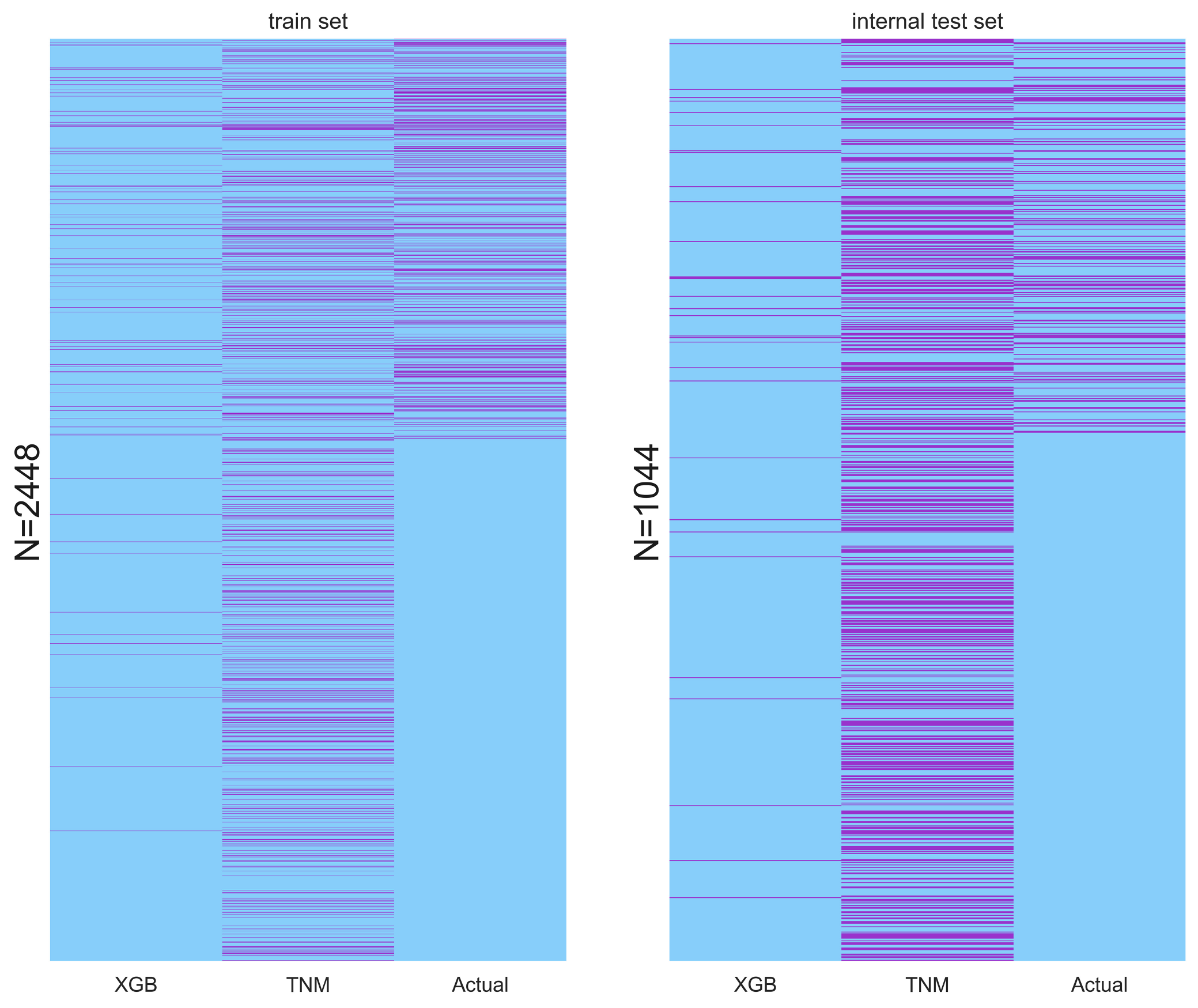

Supplement: Supplementary file 2 — Supplementary Figure S2. [file 41598_2023_45438_MOESM2_ESM.tif]
