# Supplementary material for: Predicting diagnosis and survival of bone metastasis in breast cancer using machine learning
Source: Sci Rep. 2023 Oct 25;13:18301. doi: 10.1038/s41598-023-45438-z (PMC10600146; doi:10.1038/s41598-023-45438-z)
Supplement: Supplementary file 3 — Supplementary Legends. [file 41598_2023_45438_MOESM3_ESM.docx]

**Figure S1** Results of correlation analysis between all variables in diagnostic **(A)** and prognostic model **(B)**. The heat map shows the correlation between the variables.

**Figure S2** The heatmaps shows the prediction results of all machine learning algorithm of the prognostic model and the actual situation of the training set and the verification set. Each column in the heatmap represents the model’s prediction of bone metastases for all patients in the dataset. Purple colors represent bone metastasis cases, whereas blue colors represent non-bone metastasis cases.
